# Supplementary figures and images for: Context-dependent activation of V1 parvalbumin interneurons enhances visual discrimination
Source: PLoS Biol. 2025 Nov 25;23(11):e3003518. doi: 10.1371/journal.pbio.3003518 (PMC12677792; doi:10.1371/journal.pbio.3003518)

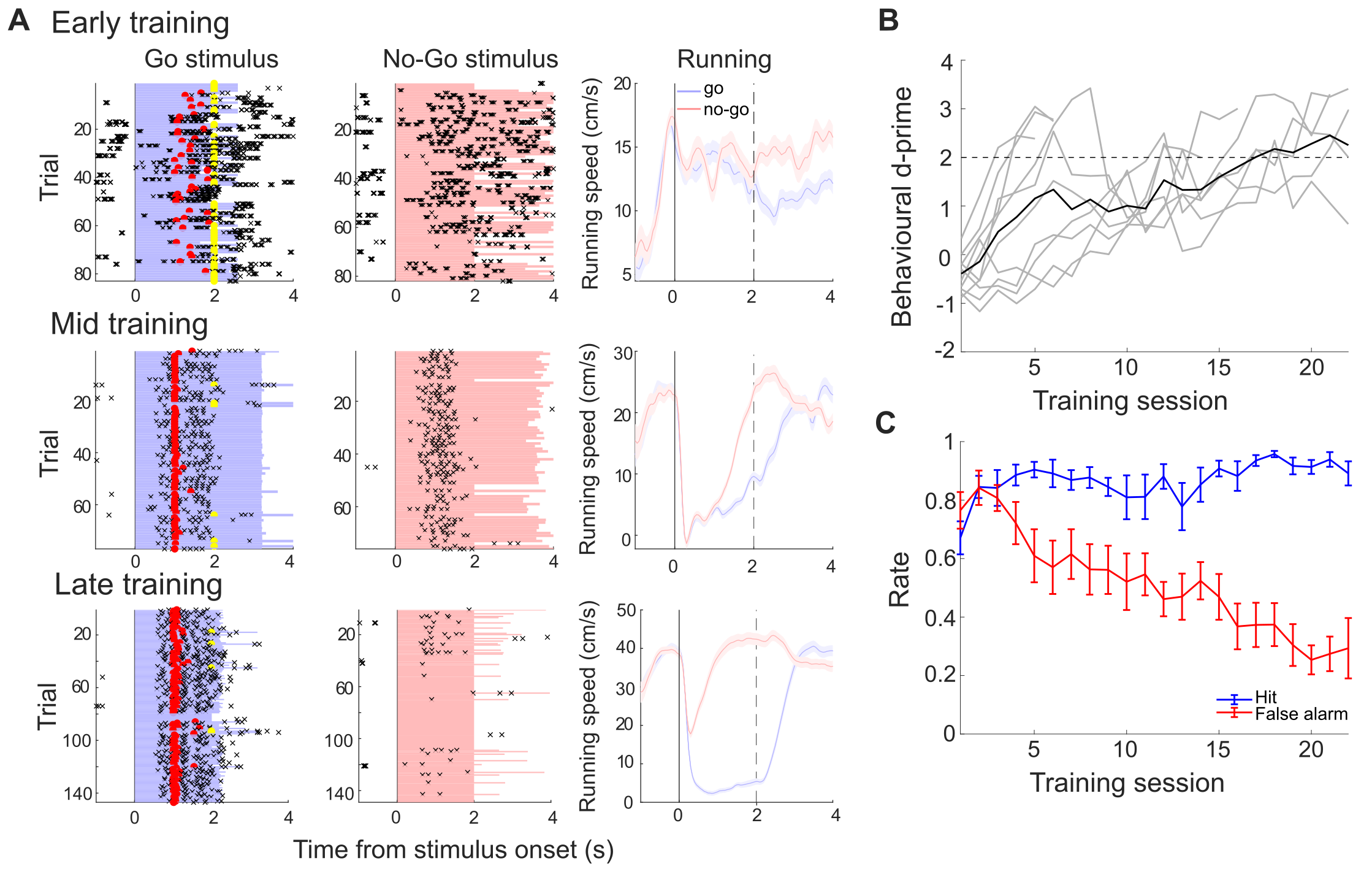

Supplement: S1 Fig — A) Changes in licking and running speed profile over training (early, mid, and late) in an example mouse. Licks (black crosses) are aligned to stimulus onset in rewarded ‘go’ trials (blue shading, left panels) and unrewarded ‘no-go’ trials (red shading, middle panels). Red dots, reward delivery triggered by licks during the response window; yellow dots, reward delivery following auto-reward trigger. Average running speed (right panels) aligned to stimulus onset, for rewarded ‘go’ and unrewarded ‘no-go’ trials for the same example sessions. Shading, SEM. B) Average behavioral performance (d’, see Methods) and C) hit and false alarm rates across training sessions. Gray lines, individual mice; error bars, SEM. (TIFF) [file pbio.3003518.s001.tiff]

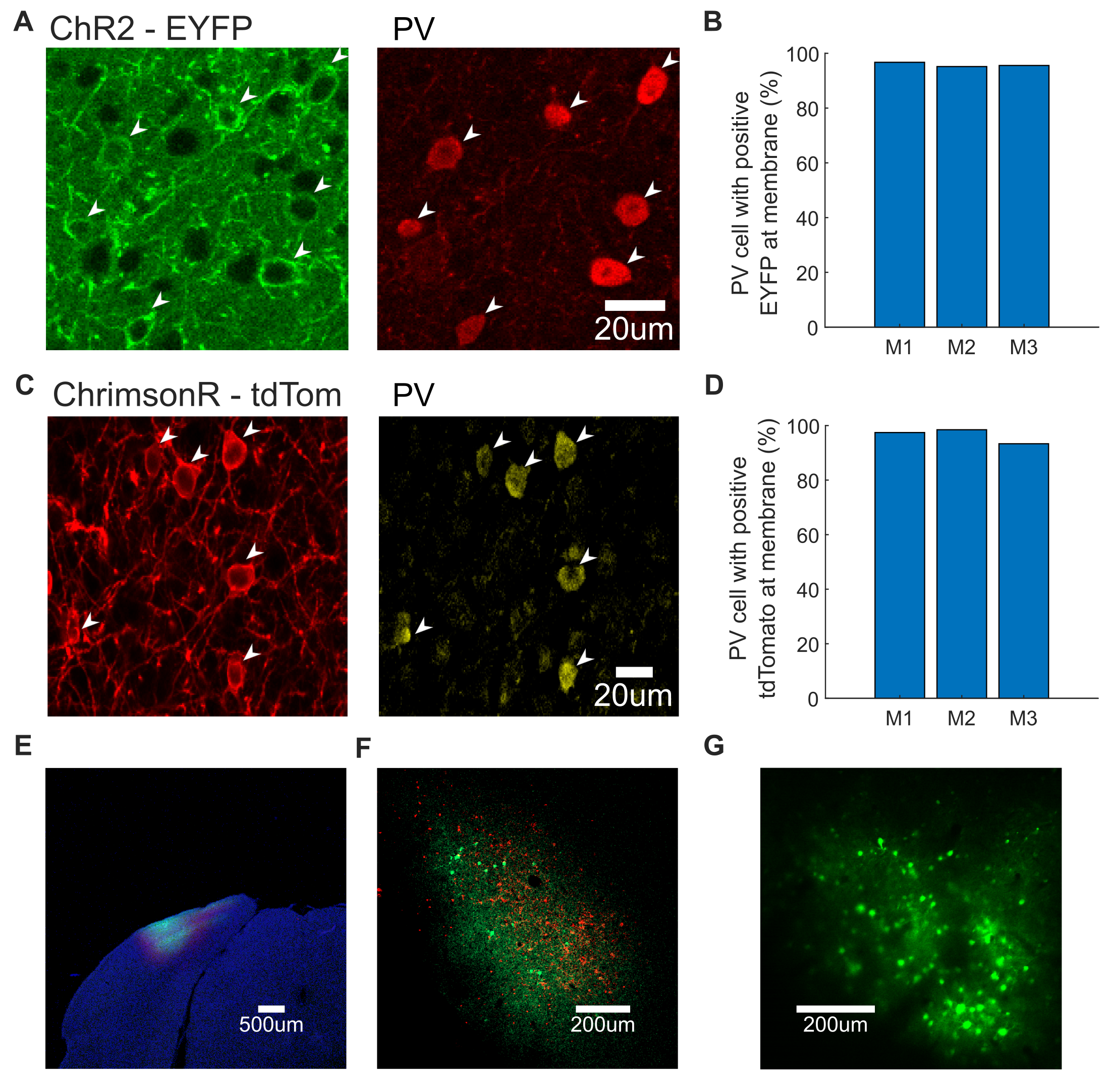

Supplement: S2 Fig — A) Example confocal fluorescence imaging region in primary visual cortex of PV-cre::Ai32 mice showing expression of ChR2-EYFP (left) at cell membrane of PV cells (right). B) Percentage of PV cells with expression of ChR2-EYFP at the cell membrane in three mice. The percentage was similar across mice: mouse 1 = 96.72% (N = 61), mouse 2 = 95.15% (N = 62), mouse 3 = 95.56% (N = 45). C) Example confocal fluorescence imaging region in primary visual cortex of PV-cre mouse at injection site, showing expression of ChrimsonR-tdTomato (left) at cell membrane of PV cells (right). D) Percentage of PV cells with expression of ChrimsonR-tdTomato at the cell membrane in three mice. The percentage was similar across mice: mouse 1 = 97.44% (N = 39), mouse 2 = 98.46% (N = 65), mouse 3 = 93.33% (N = 30). White arrows indicate identified PV cells. E) Confocal microscope image (2.5x objective) of coronal slice showing GCaMP (green) and ChrimsonR (red) overlaid on DAPI stain (blue) showing labeling in V1 across cortical layers 1–6 (with injections at 200 and 400 micrometer). F) Example confocal image of horizontal slice showing GCaMP (green) and ChrimsonR (red) fluorescence. Full width at half maximum (FWHM) estimates of spread of fluorescence were similar for GCaMP (median 974 micrometer, 738–1,028, N = 5) and ChrimsonR median (763, 733–1,010, N = 5). G) Example zoomed out in-vivo two-photon microscope image (imaging of neuronal activity was done with increased zoom). Labeled cell bodies were contained within area of ~800 micrometer (N = 4 examples). (TIFF) [file pbio.3003518.s002.tiff]

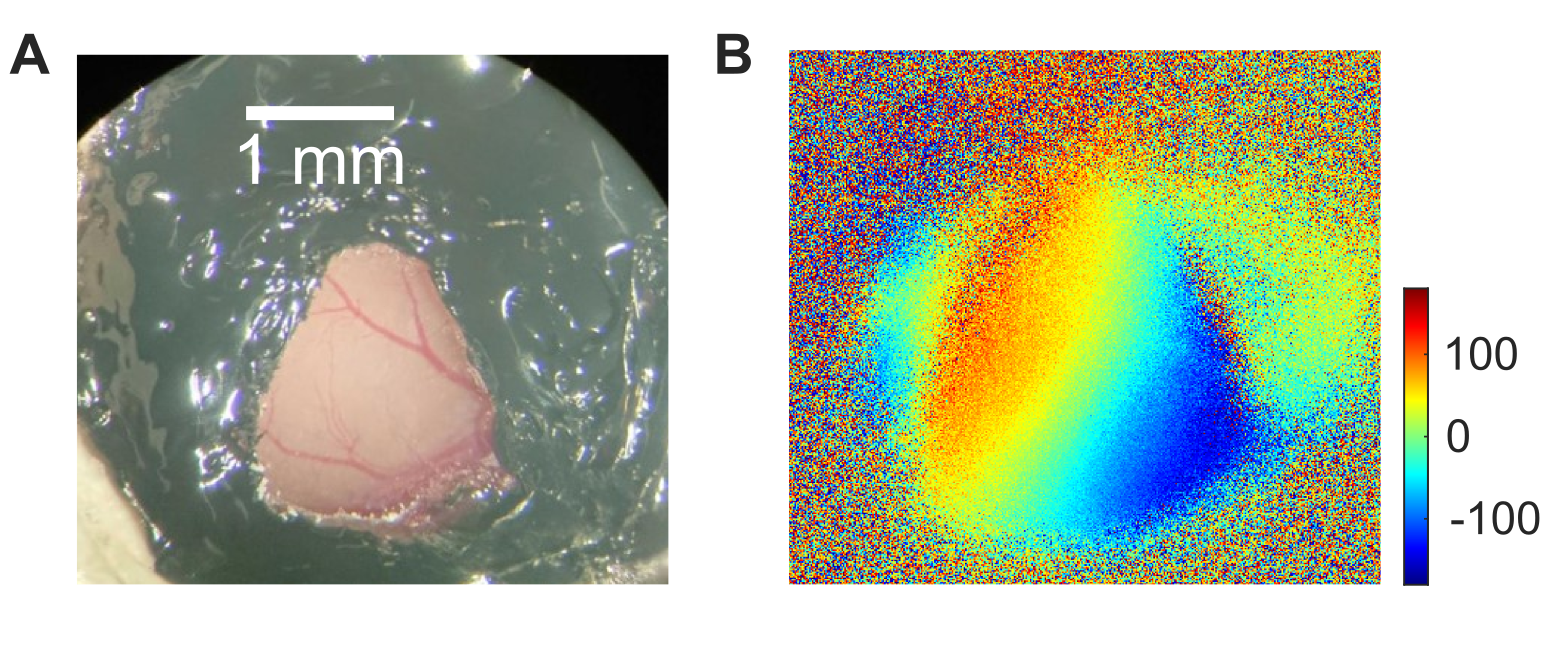

Supplement: S3 Fig — A) Example mask aligned to anterior margin of transverse sinus and stereotaxic coordinates to optogenetically stimulate primary visual cortex (see Fig 1F showing abolished visual performance with high power in all mice). B) Example retinotopic map using intrinsic imaging [7,93]. Colourscale indicates −180 (monocular) to 180 (binocular region) degrees spanning 110 visual degrees covered by monitor. (TIFF) [file pbio.3003518.s003.tiff]

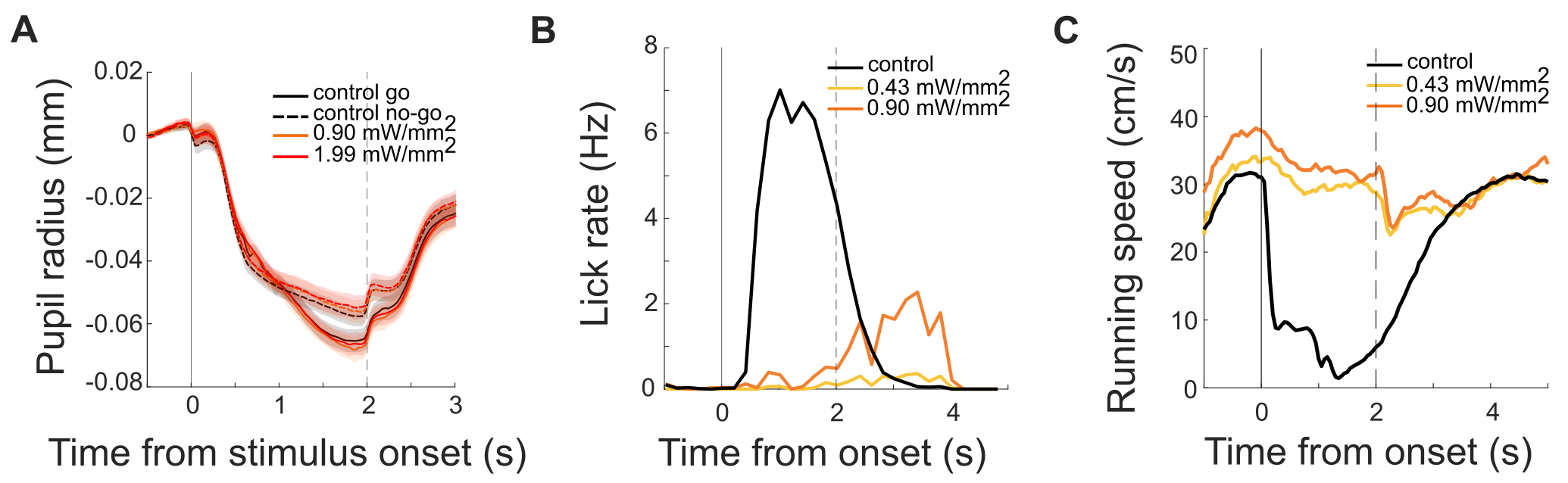

Supplement: S4 Fig — A) Average pupil size across different laser powers (including 0 mW/mm2) in ‘go’ (continuous line) and ‘no-go’ (interrupted line) conditions in WT mice. Shading, SEM. B) Lick rate during the presentation (0–2 s) of stimulus only (black) or laser stimulation only (color) in PV-ChR2 mice. Proportion of licks during the baseline period, −1–0 s, was compared to the proportion of licks during the laser stimulation period, 0–2 s, using a Chi-square test and no significant difference was detected in both laser conditions. C) Running speed aligned to stimulus onset (black) or laser stimulation onset (color). Based on their licking and running behavior, mice cannot detect the laser stimulation alone during the 2 s period in the absence of visual stimulus. Vertical continuous line, stimulus or laser onset; vertical interrupted line, stimulus or laser offset. (TIFF) [file pbio.3003518.s004.tiff]

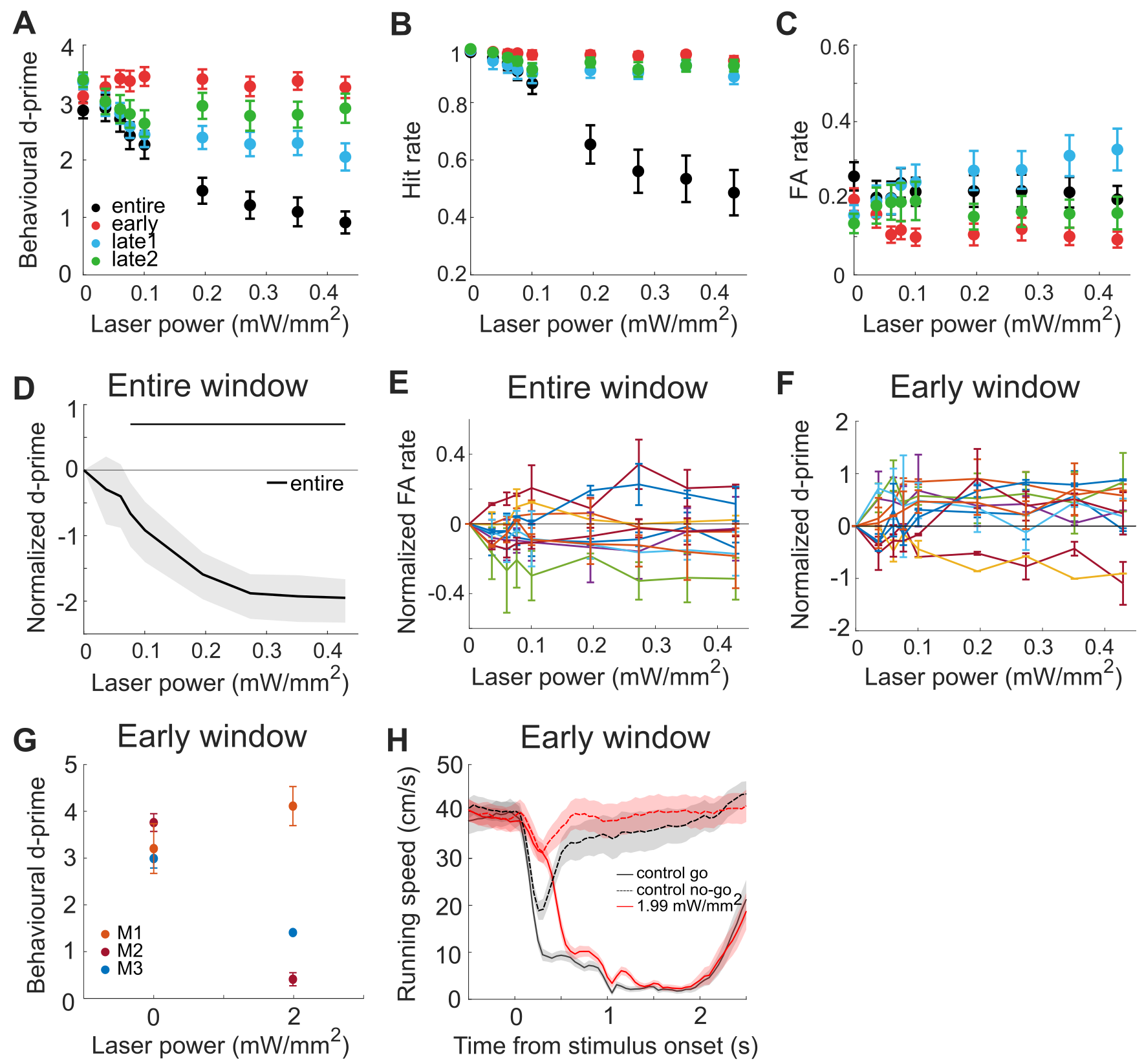

Supplement: S5 Fig — A) Average discrimination performance, d’, B) hit rate, and C) false alarm rate for all time window conditions. D) Normalized average discrimination performance, d’, in the ‘entire’ window for sessions with lower hit rate (> 0.95). Shading, 95% CI of bootstrap distribution. Data is normalized by subtracting the value of the 0 power level during 0–2 s of stimulus onset. The same normalization method is applied across all normalized data plots regardless of the stimulation window duration. E) Normalized false alarm rate in the ‘entire’ window. Color lines, individual mice (N = 10, 41 sessions). F) Normalized discrimination performance, d’, in the ‘early’ window. Color lines, individual mice (N = 10, 37 sessions). G) Average discrimination performance, d’, in three mice, where high laser power (2 mW/mm2) was used to silence the ‘early’ window (6 sessions). As expected, two of three mice showed impaired performance. H) Average running speed for ‘go’ (continuous) and ‘no-go’ (interrupted) conditions for the mouse which retained high performance when the ‘early’ window was silenced at 2 mW/mm2. Running was delayed relative to stimulus presentation. In all plots, except D), error bars or shading indicate SEM. (TIFF) [file pbio.3003518.s005.tiff]

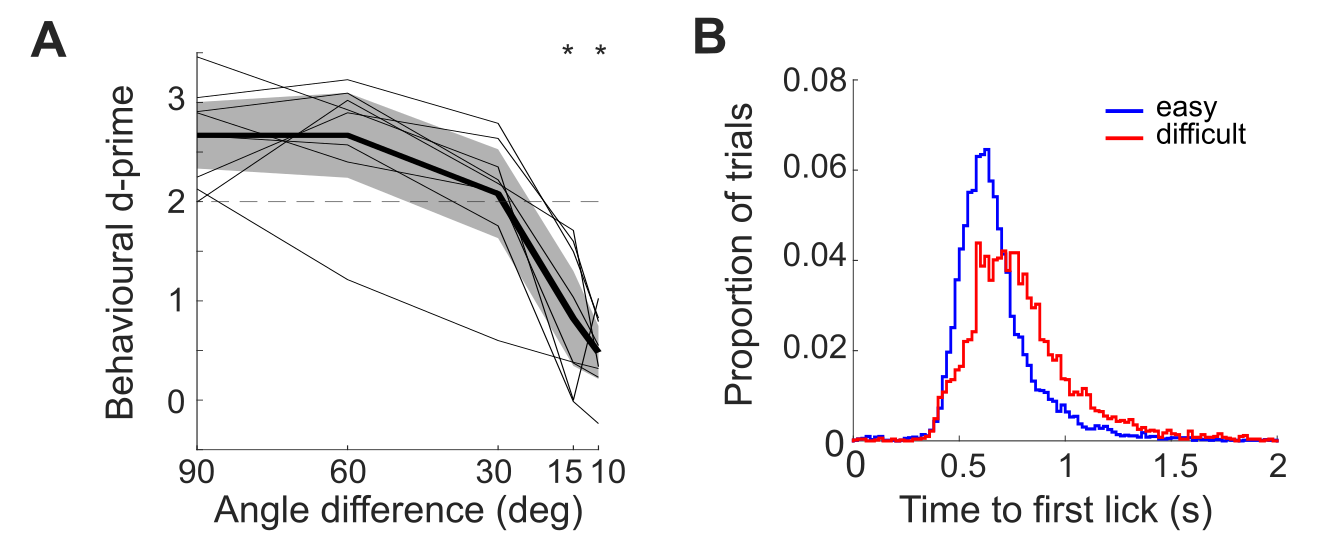

Supplement: S6 Fig — A) Behavioral d’ across the go/no-go discrimination task for the trained (90° angle difference) and test (60°, 30°, 15°, and 10° angle difference) orientations (Wilcoxon signed-rank test, 15° p = 0.031, 10° p = 0.031, after Bonferroni correction, N = 8 mice). B) Response latency distributions (20 ms bins) across different task difficulty levels, measured by the time to first lick. 25th and 75th percentiles are 550–740 ms for easy (90° angle difference), and 630–900 ms for difficult (15° angle difference) discriminations. (TIFF) [file pbio.3003518.s006.tiff]

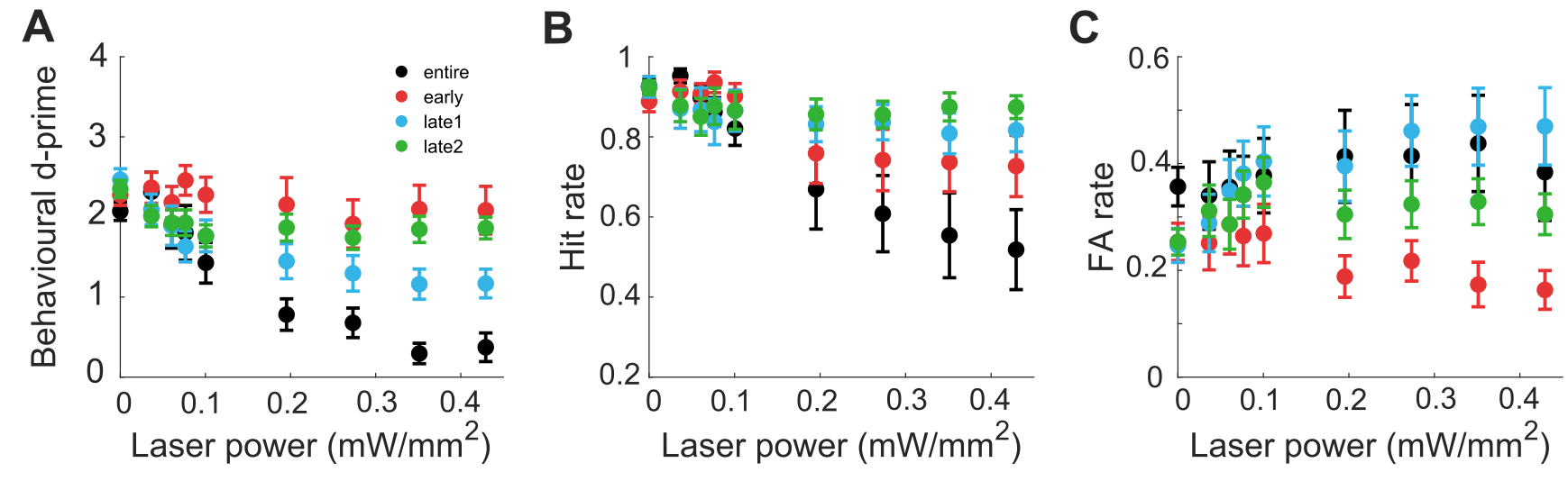

Supplement: S7 Fig — A) Average discrimination performance, d’, B) hit rate, and C) false alarm rate as a function of laser power for all time window conditions in the difficult task. Error bars, SEM. (TIFF) [file pbio.3003518.s007.tiff]

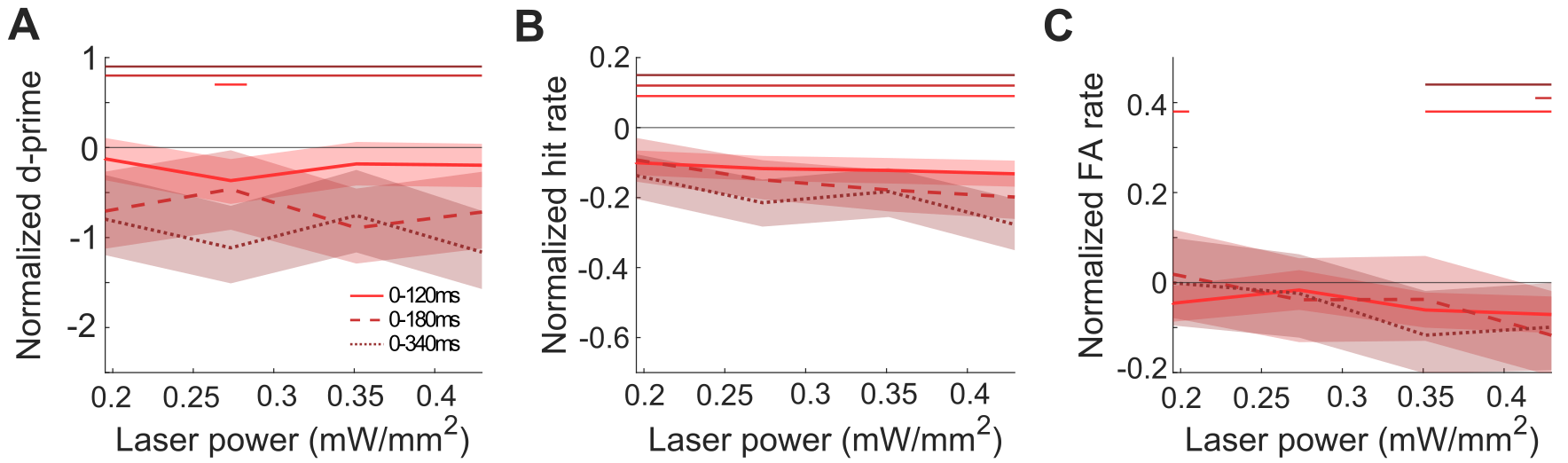

Supplement: S8 Fig — A) Normalized average discrimination performance, d’, B) hit rate, and C) false alarm rate as a function of laser power for the original (0–120 ms) and two new (0–180 ms and 0–340 ms) ‘early’ windows (N = 4 sessions). Data is normalized by subtracting the value of the 0 power level (see Methods). Shading, 95% CI of bootstrap distribution. (TIFF) [file pbio.3003518.s008.tiff]

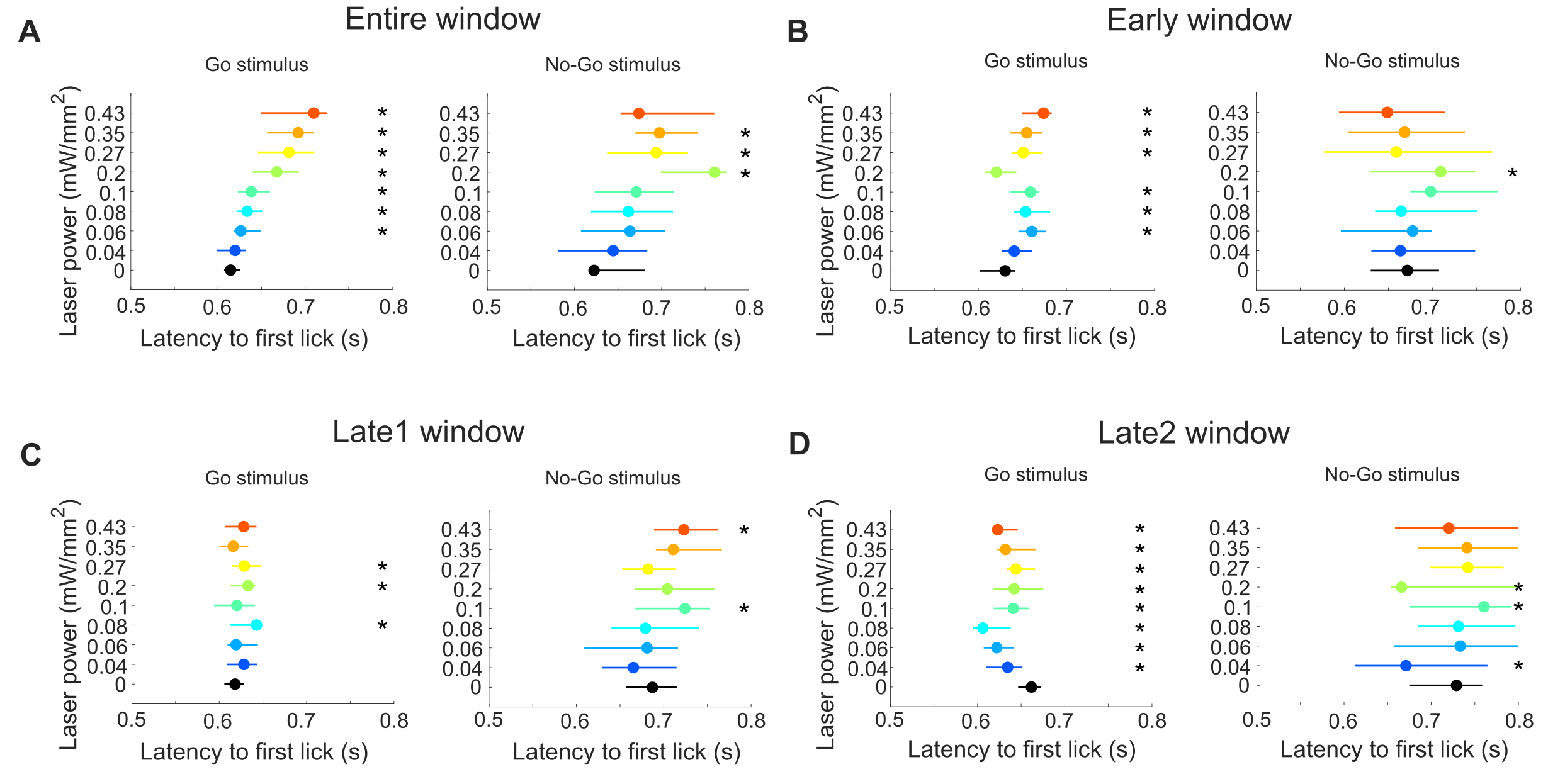

Supplement: S9 Fig — Response latency for time to first lick in laser off (0 mW/mm2) versus laser on (0.04–0.43 mW/mm2). Circle, median; line, 95% CI of bootstrapping. Asterisks indicate significant deviation (p < 0.05) from no laser stimulation control condition. The number of mice and sessions is the same as in Fig 2. (TIFF) [file pbio.3003518.s009.tiff]

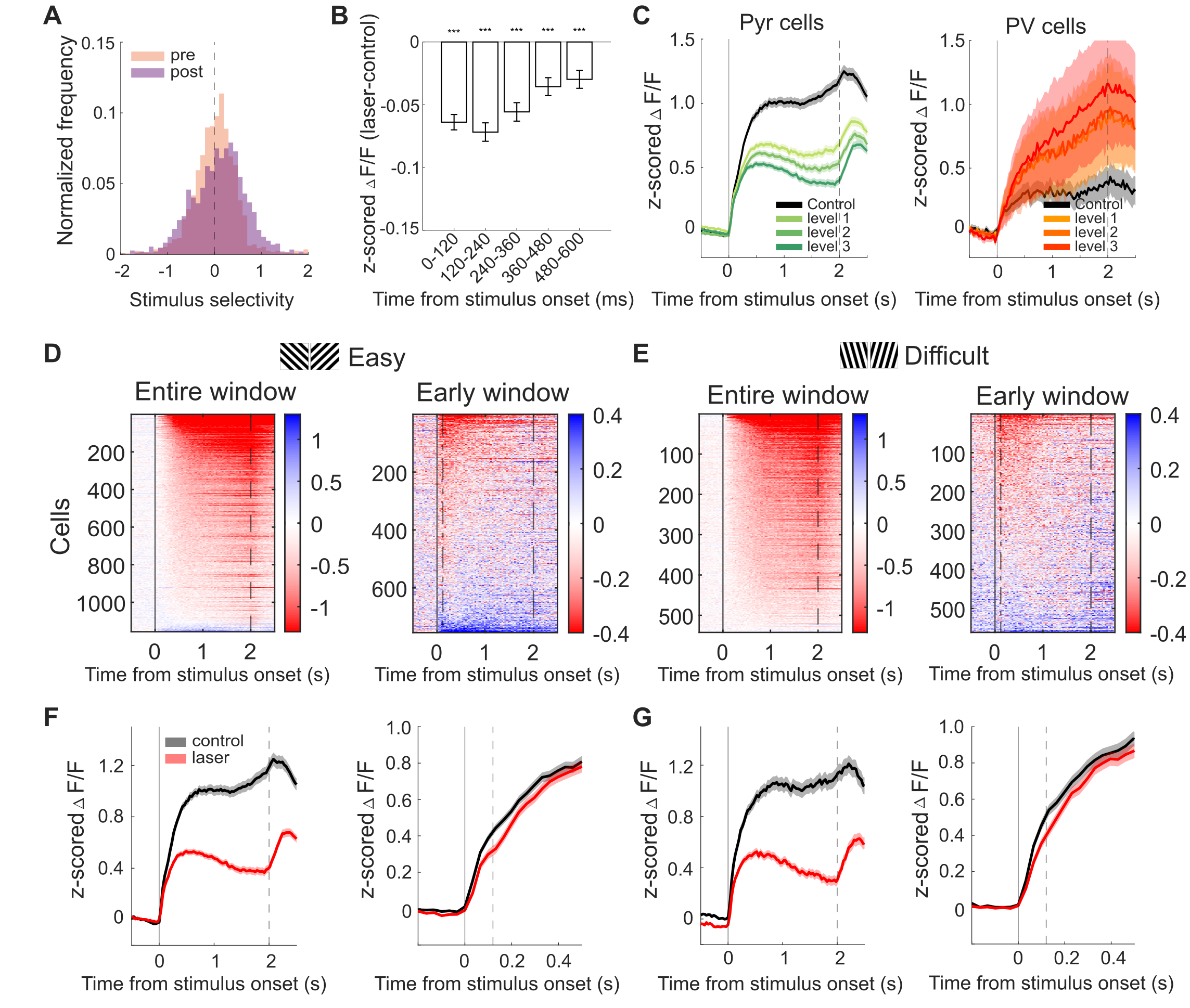

Supplement: S10 Fig — A) Histogram of stimulus selectivity (positive values: cells prefer rewarded go stimulus; negative values: cells prefer unrewarded no-go stimulus) for pre- and post-learning of the visual discrimination task. Pre: n = 1,147, 10 mice, 0.05 ± 0.02; Post: n = 1,467, 9 mice, 0.14 ± 0.02; p < 0.002 (Wilcoxon rank-sum test). Median ± SEM. B) Average stimulus response activity at different times, ranging from 0 to 120 ms (laser duration), 120–240, 240–360, 360–480, and 480–600 ms, during the early window stimulation (0–120 ms) when stimulating PV cells with highest laser (control subtracted). Error bars, SEM. C) Average responses of two population of neurons (left panel, Pyr cells; right panel; PV cells) to optogenetic laser illumination at three different levels during the entire stimulus duration (0–2 s). The activity to the visual stimulus of Pyr cells (n = 1,156) is suppressed, whereas PV cells (n = 39) is increased. Shading, SEM. D) Difference in mean visual stimulus-evoked response with PV cell activation (control subtracted), aligned to visual stimulus onset (continuous line). Cells were ordered by their averaged activity at 0–1 s. Color bar range indicates activity, with positive values (blue) for cells increasing their activity and negative values (red) for cells decreasing their activity as a result of laser stimulation. E) is same as D) but for difficult task. F) Average activity response to visual stimulus in the absence (black) and presence (red) of laser stimulation, for the entire (left panel) and the early window condition (right panel). G) is same as F) but for difficult task. In plots D) to G): dashed line at 2 s for the entire window and at 0.12 s for the early window marks the laser stimulation offset; data shown for the highest laser power. (TIFF) [file pbio.3003518.s010.tiff]

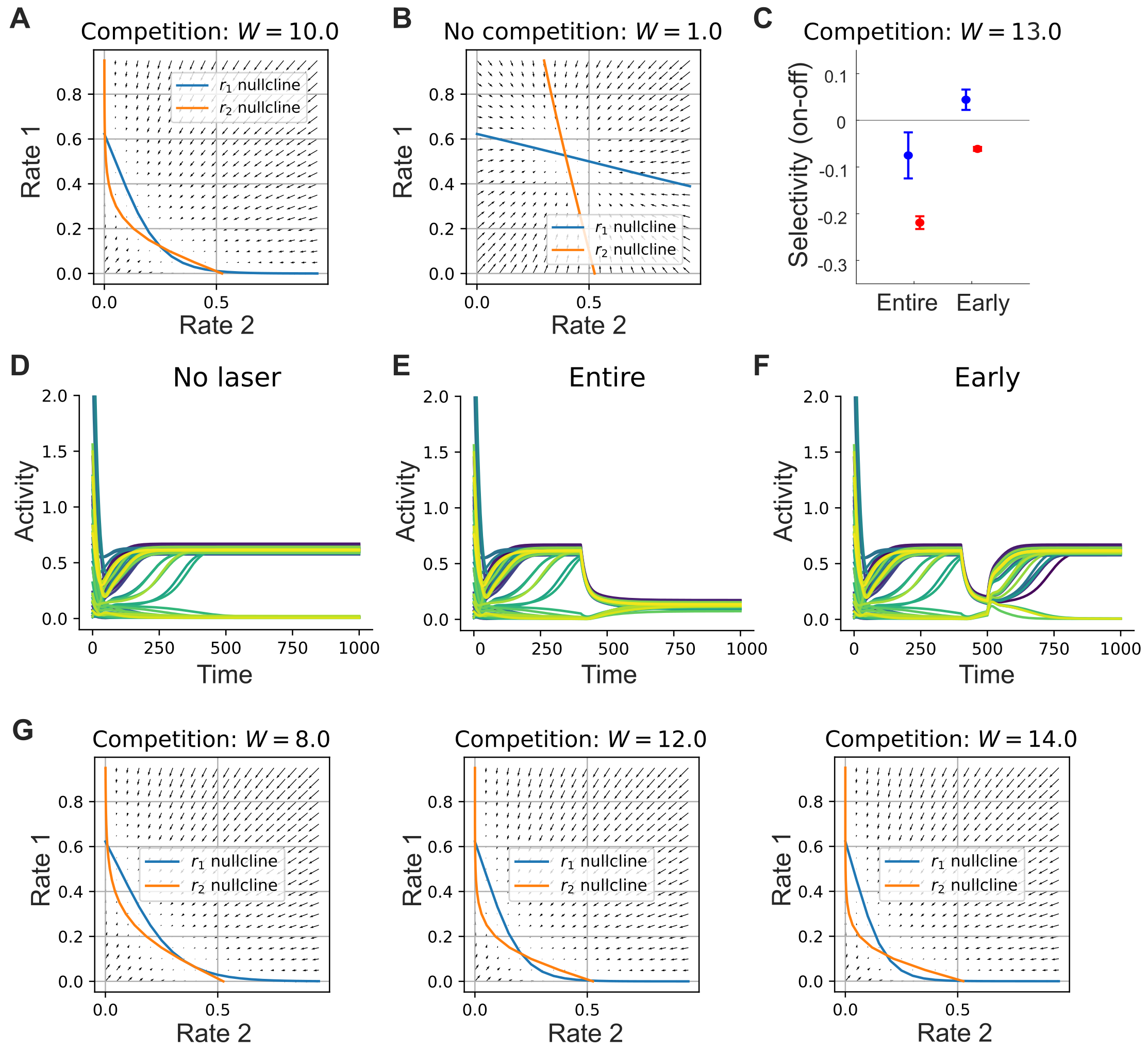

Supplement: S11 Fig — Phase planes for A) a circuit with strong competition (W=10.0) and B) a circuit with no competition (W=1.0), when the preferred stimulus of population 1 is present and the laser is off (I1=0.5; I2=0.1;PV=0). The r1 nullcline is shown in blue and the r2 nullcline is shown in orange. For a circuit with strong competition, there are two stable fixed points, where either the rate of population 1 r1 or the rate of population 2 r2 is large. For a circuit with weak competition, there is only one fixed point. C) The difference in selectivity between laser on and laser off conditions for go- (blue) and no-go-preferring (red) populations for ‘Entire’ and ‘Early’ PV cell activation. Competition in the circuit has been increased to W=13.0, demonstrating that the conclusions were not strongly dependent on precise parameter choices (see also G). Firing rates over time for 15 simulations of go-preferring populations during 50 go trials with either D) no laser stimulation, E) entire laser stimulation, or F) early laser stimulation. In the no laser condition, the activities end up in one of two fixed points. In the early laser stimulation, most activities end up in the same fixed point. G) Phase planes for a circuit with different competition weights varying from 8.0 to 14.0. The r1 nullcline is shown in blue and the r2 nullcline is shown in orange. There are two stable fixed points, where either the rate of population 1 r1 or the rate of population 2 r2 is large. (TIFF) [file pbio.3003518.s011.tiff]
