# Supplementary material for: Context-dependent activation of V1 parvalbumin interneurons enhances visual discrimination
Source: PLoS Biol. 2025 Nov 25;23(11):e3003518. doi: 10.1371/journal.pbio.3003518 (PMC12677792; doi:10.1371/journal.pbio.3003518)
Supplement: Attachment — Submitted filename: ResponseToReviewers.docx [file pbio.3003518.s014.docx]

# Reviewer #1:

The role of PV cells shaping visual perception is murky. This is an important area of investigation. The authors attempt to tease out the relation between PV cell activity and visual perception via a behavioral go/no-go licking task performed while optogenetically activating PV cells with different intensities of laser stimulation, use 2P imaging to assess cell selectivity under different conditions, and propose a model to explain their findings. While the authors do show that behavioral measures are differentially impacted by optogenetic activation of PV cells occurring in an early window during visual stimulation compared to later activations, and depend on the difficulty of the task, the 2P data is much weaker as presented (due largely to the decision to show only highly-processed results) and the model is sufficiently abstract/unmotivated that it is hard to credit it as capturing the underlying biology in a meaningful way.

We thank the reviewer for their supportive comments and helpful comments and suggestions.

Specific responses follow in roughly chronological order:

* What percent of PV cells are infected by ChR2? Is this consistent across animals and, if not, how are you controlling for this in your analysis and data interpretations? There is no histology or reporting of these types of numbers which can be important for assessing experimental methodology and comparison with other papers.

We thank the reviewer for this important question. We have now added immunohistological analysis to determine the percentage of PV+ cells that are positive for ChR2 (Supplementary Fig. S2A-B). In 3 PV-Cre::Ai32 mice the average percentage of PV cells that were also positive for ChR2 was 95.83 % (N=168 cells). This percentage was similar across mice: mouse 1: 96.72 % (N=61), mouse 2: 95.15 % (N=62), mouse 3 95.56 % (N=45). This is consistent with previous reports using the PV-cre transgenic mouse lines (Pfeffer et al., 2013, Nat Neurosci; Hippenmeyer et al., 2005, PLoS Biol; Nocon et al., 2023, Commun Biol). We have updated the text in section ‘Strong activation of PV cells in V1 impairs discrimination performance’.

* The "early" optogenetic stimulus case could cause rebound spiking in the late period that would interfere with task performance. This general effect has been reported in different circumstances and can have a real impact on network activity, even in some cases initiating epileptic activity via rebound spiking in pyramidal cells (Sessolo et al. J Neuro 2015). The authors should determine the extent to which this is happening in their data and whether it could account for some of their findings that differentiate the early and late stimulation cases.

We thank the reviewer for this comment. To investigate the possible rebound effect, we compared the difference between the control (laser off) and the laser on condition (highest laser) at different times during stimulus presentation, ranging from 0-120 (laser duration), 120-240, 240-360, 360-480, and 480-600 ms. In all these windows we observed a reduction of activity in the laser on condition compared to the control condition, indicating the laser offset at 120ms did not evoke an increased response. We added this panel to Supplementary Fig. S10B. Additionally, we have added a reference to the text in section ’Changes in neural selectivity reflect behavioural performance in easy but not difficult discriminations’ discussing the conditions under which rebound effects are likely to play a role: these effects are strongest with longer duration of stimulation and higher powers (see Li et al 2019). Importantly, in our data we find consistent behaviour improvements for the easy condition even with relatively low laser powers and with a very short duration of stimulation.

Average stimulus response activity at different times, ranging from 0 – 120 ms (laser duration), 120 – 240, 240 – 360, 360 – 480, and 480 – 600 ms, during the early window stimulation (0 – 120 ms) when stimulating PV cells with highest laser (z-scored ∆F/F, laser on - control). Error bars, SEM.

Fig 1S E is interpreted to show that mice cannot detect laser stimulation alone based on an absence of licking within the first 2 seconds. There is, however, a clear increase in licking after 2 seconds for the higher laser power (0.9 mW/mm2). This is hard for me to interpret because it isn't reported which laser stimulus protocol is being used in this figure (I assume it's either entire or one of the lates) but assuming that the laser is on for 2 sec, it does seem to show that there is a behavioral consequence of rebound activity when the PV block is released. It would be surprising if mice licked to a sudden absence of visual perception as PV cells activated when they had been trained to lick only when presented with a specific target orientation. The fact that the mice do start to lick after the laser turns off does not accord well with the broader conclusions from this panel and is potentially interesting if it indicates a "rebound perception" of the target grating- in essence activating what you describe as a "stimulus-selective ensembles with Pyr neurons" on page 5 in a manner sufficient to drive behavior. I'm also not sure how to reconcile this with the conclusion that mice adopt a more cautious strategy in the presence of stimulation.

We thank the reviewer for this comment. In the control plot of Fig S1 E (now Fig. S4B), we are using the entire window stimulation (0 - 2 s of laser stimulation), which we have now clarified in the manuscript. While we agree that there is an increase in licking following the stimulation offset, as the reviewer has pointed out, this is evident only in the higher laser power (0.9 mW/mm2). This power was only used for this control experiment (Fig. S4B-C). The maximum power that was used in behavioural testing across all plots was 0.43 mW/mm2, and importantly this power does not induce licking following the offset. All other powers used for behavioural testing were lower than 0.43 mW/mm2. Moreover, we analyse performance by only taking the licks occurring during the 0 - 2 s window (onset to offset of visual stimulus). We do not include any licks beyond the stimulus offset at 2s, therefore even if any licks are induced by laser stimulation, these would not affect our results or conclusions. Another important point is that behavioural improvements in the early window stimulation are mainly caused by a reduction of false alarm rate. Thus, high stimulation levels increasing licking would likely cause increases in both hit and FA rates, which is not seen in our data.

The reviewer also asks how to reconcile the licking with the highest power level with the conclusion that mice adopt a more cautious strategy in the presence of stimulation. Similarly, in Fig. 4B all behavioural analysis is done on the entire stimulus duration (0 - 2 s), and not beyond. Any changes that PV cell activation causes on the decision criterion are only quantified during the 0-2s period. Therefore, Fig. 4 showing the decision criterion increases, indicating that mice become more conservative in producing licks to any visual stimulus, is not in contrast to the control plot for two reasons. Fig. S4B shows no significant increase in licks during the 0 - 2 s period, and all tested powers are within 0 to 0.43 mW/mm2.

* Fig S2 and it's caption need some work for clarity. For example, what does "normalized average false alarm rate in the 'entire' window" mean compared to the 'early' window mean? This wording seems to suggest that the false alarm rate was calculated based on licking activity during the period of laser stimulation which is not what I think is actually meant based on the methods. It would also help to put title labels on these plots so that I don't have to keep going back and forth to the caption to know what is shown in each panel (same true in other figures too).

We agree with the reviewer that the original wording was ambiguous. Indeed, as the methods section suggests, we calculated d’, hit and FA rate by always taking the responses during 0 - 2 s regardless of the duration of optogenetic stimulation. Normalizing the data is therefore done by subtracting the value in the control condition (0 laser power) during 0 - 2 s of stimulus onset from the corresponding laser condition. We have added this now to the figure legend. We have also added title labels to the plots in this and other figures.

Figure S5: Changes in behavioural readouts as a function of laser power in the easy discrimination task. A) Average discrimination performance, d’, B) hit rate, and C) false alarm rate for all time window conditions. D) Normalized average discrimination performance, d’, in the ‘entire’ window for sessions with lower hit rate (> 0.95). Shading, 95% CI of bootstrap distribution. Data is normalized by subtracting the value of the 0 power condition during 0 - 2 s of stimulus onset. The same normalization method is applied across all normalized data plots regardless of the stimulation window duration. E) Normalized false alarm rate in the ‘entire’ window. Coloured lines, individual mice (N = 10, 41 sessions). F) Normalized discrimination performance, d’, in the ‘early’ window. Coloured lines, individual mice (N = 10, 37 sessions). G) Average discrimination performance, d’, in three mice, where high laser power (2 mW/mm2) was used to silence the ‘early’ window (6 sessions). As expected, two of three mice showed impaired performance. H) Average running speed for ‘go’ (continuous) and ‘no-go’ (interrupted) conditions for the mouse which retained high performance when the ‘early’ window was silenced at 2 mW/mm2. Running was delayed relative to stimulus presentation. All plots, except D), error bars or shading indicate SEM.

* Fig 3, I agree that your data provides performance-based evidence that mice are better at discriminating 90° than 15°, but there are reports that mice can reliably discriminate at 9° difference (Lyamzin et al PNAS 2021) or even lower. More discussion/analysis of the extent to which this task is actually hard as opposed to simply harder, and what is known about PV cells' contributions to this, would be appropriate and help interpret overall conclusions.

We thank the reviewer for this suggestion. When developing the task, we tested a range of orientations by gradually reducing the angle difference between the ‘go’ and ‘no-go’ gratings to as little as 10 degrees. We tested 8 mice to identify a difference at which they showed significantly reduced performance but were still able to reliably discriminate. We found that stimuli separated by 15 degrees were an appropriate choice, consistent with previous literature (Lee et al., 2012; Resulaj et al., 2018), because mice showed a significantly reduced performance. We trained mice on the new stimuli until they achieved d’ > 1.5 (with average performance during testing d’ > 2, Fig. S6A). To further identify whether mice found discriminating 15° difficult, we plotted the distribution of their reaction times (time to first lick). Interestingly, the latency distribution for our go/no-go task resembled the human reaction time distribution (Alais et al., 2016, Scientific Reports) – difficult discriminations had a broader distribution with longer latencies between 630 to 900 ms, compared to easy discriminations with latencies in the range of 550 to 740 ms (for 25th and 75th percentiles) (Fig. S6B). We have now added these plots to a Supplementary figure, and have added explanation for our choice of 15° angle difference to the main text in section ’Difficult discriminations do not benefit from PV cell activation’.

We are not aware of studies that directly investigate the role of PV cells during easy versus difficult discriminations, but we have included a section about the orientation tuning of PV cells potentially restricting their contribution to fine discriminations in the discussion.

Figure S6; related to Figure 3. Testing a difficult discrimination task. A) Behavioural d’ across the go/no-go discrimination task for the trained (90° angle difference) and test (60°, 30°, 15°, and 10° angle difference) orientations (Wilcoxon signed-rank test, 15° p = 0.031, 10° p = 0.031, after Bonferroni correction, N = 8 mice). B) Response latency distributions (20ms bins) across different task difficulty levels, measured by the time to first lick. 25th and 75th percentiles are 550 to 740 ms for easy (90° angle difference), and 630 to 900 ms for difficult (15° angle difference) discriminations.

* Fig 4, is there a latency difference between the late1 and late2 cases? The authors use latency to show that the mice don't seem to be using a strategy of waiting to accumulate more evidence in the "early" case, but it's not clear what the distribution of lick times looks like for the late1 and late2 cases. Given that there is only a 60 ms difference between the two cases, it's surprising that they separate as much as they do and I'd like to see if there is any delay to lick created by the late stim cases.

We initially focused our analysis on the early window, where we saw the behaviour improvement, to determine whether this effect could be explained by a speed-accuracy trade-off. We concluded this was not the case since the improvement came from a reduction of false alarm rates but the latency for false alarms was not shorter for higher laser powers. Therefore we concluded that the data was not consistent with mice waiting longer to accumulate more evidence.

As the reviewer indicates, there is a clear separation between the discrimination performance (Fig. 2E) in the late1 (starting at 120ms) and late2 conditions (starting at 180ms, 60ms later). This separation is explained by the difference in the false alarm rate (Fig. 2G) which increases with increasing laser power. We therefore determined the lick latencies during these two conditions. If the reduced performance was due to a speed accuracy trade-off (where the mouse gets more accurate when it waits longer, and vice versa), we would expect that mice would show faster responses to the no-go stimulus with increasing laser power (particularly in the late1 condition where performance is poorer). However, we see that this effect is not present. We have now added these panels to a Supplementary Fig. S9 related to Figure 4.

Figure S9; related to Figure 4. Effects of PV cell activation on the lick latency of go and no-go stimuli across the different time windows in the easy discrimination task. Response latency for time to first lick in laser off (0 mW/mm2) versus laser on (0.04 – 0.43 mW/mm2). Circle, median; line, 95% CI of bootstrapping. Asterisks indicate significant deviation (p < 0.05) from no laser stimulation control condition. The number of mice and sessions is the same as in Figures 2.

Fig 4A - x label should be more descriptive, maybe "latency to first lick"

We have modified as suggested.

Fig 4B, I don't understand the stats - this is not normalized as done previously so I can't use 95% CI around zero to see how the data differs from the null case directly. Normalized to match your previous procedures or a plot of the non-laser data would help with transparency on this stat.

As the reviewer suggested, we have now normalised the plots similar to the other figures and updated Fig. 4B and accompanying text and method descriptions. With both methods, we see that PV cell activation affects the decision criterion dependent on laser power. We have updated the text in section ‘PV cell activation affects decision criterion’.

B) Normalized decision criterion c across time windows in easy (left panel) and difficult (right panel) discriminations. Shaded line is the 95% CI of data shuffled 1000 times, using bootstrapping with replacement. Horizontal bars with corresponding colours indicate significant deviation (p < 0.05) from no laser stimulation. Data is normalized by subtracting the value of the 0 power level (see Methods).

Fig 4B, it's notable that the "entire" case is so different from the other cases. Worthy of a comment and some speculation as to why this is so. Are they waiting later

Indeed, the entire condition is different from other conditions because it shows a much greater change in the decision criterion. Positive shifts indicate that the mice become more conservative in their responses. However, this is not necessarily linked to lick latency. While significant increases in the lick latency are detected (Supplementary Fig. S9A), these are only present when animals respond to the go stimulus. Furthermore, we also noticed that there were conditions with no increase in criterion that still had a significant change in lick latency (Supplementary Fig. S9). We have added discussion to the text.

* There is so little 2P data shown that it's hard to know what to make of it. Figure 5 shows only average z-scored data from only 2 example cells out of 754 that went into the data set. There are no supplemental figures showing what raw data looked like in any way leaving me with no ability to independently assess the GCAMP expression levels, how much of the population was responding to stimulation, the extent to which the dynamic responses to stimulation in these 2 cells are representative of the broader population, the percent of cell that were selective and the heterogeneity of PV modulation across this population, where imaging was occurring relative to V1m/b boundaries, etc. Even ignoring this and accepting the data & conclusions as presented, I'm still confused by these results since only the easy task shown a significant effect of PV activation on selectivity even though stimulation had an effect on behavior measures show previously. I would like to see how cellular activation dynamics (as in panel C) differ when the "early" laser activation is used and how they differ for easy and hard stimulus classes. This figure is asking me to take way too much on faith and presenting the data in such a processed/sanitized form that I can't assess its validity or gain any insight into neural activity beyond the one specific metric the authors focus on.

We thank the reviewer for the comment. We have now included an additional Supplementary Fig. S10 containing plots with raw data. We have updated the text in section ‘Changes in neural selectivity reflect behavioural performance in easy but not difficult discriminations’. Several points were raised, which we address individually below:

Q: Assess the GCAMP expression levels.

Response: We have now added description of the horizontal and laminar spread of GCaMP expression (Fig. S2E-G).

Q: How much of the population was responding to stimulation.

Response: We have quantified the percentage of cells that shows a significant response to the laser. Out of 1156 Pyr cells, 965 of those cells were significantly modulated by the PV cell activation, accounting for 83.48 %.

Q: Extent to which the dynamic responses to stimulation in these 2 cells are representative of the broader population.

Response: We have now added the PSTH for the averaged population response (Fig. S10C), and we show that the example 2 cells are representative of the broader population.

Q: Percent of cell that were selective and the heterogeneity of PV modulation across this population.
Response: We have now also quantified the percentage of cells showing a preference for the go or no-go stimulus and we have added the distribution of PV modulation. Out of 1156 Pyr cells, 630 of those cells have a bias to the go stimulus and were significantly modulated by the laser, accounting for 54.50 % (344 cells are significantly selective to the go stimulus and significantly modulated, accounting for 29.76 %). Out of 1156 Pyr cells, 335 of those cells have a bias to the no-go stimulus and were significantly modulated by the laser, accounting for 28.98 % (157 cells are significantly selective to the no-go stimulus and significantly modulated, accounting for 13.58 %).

Q: Where imaging was occurring relative to V1m/b boundaries.

Response: Injections were made at 2.5 and 2.3 ML corresponding to the monocular segment of the primary visual cortex (Wang and Burkhalter, 2007). Before two-photon recordings, we confirmed that cells in our imaging field were visually responsive to stimuli presented in the monocular visual field (at the center of the screen positioned at 45 degrees relative to the body axis of the mouse).

Q: How cellular activation dynamics (as in panel C) differ when the "early" laser activation is used and how they differ for easy and hard stimulus classes.

Response: We have now added plots showing the average difference between the control and the laser for all cells. We have also now shown the averaged activity for the control versus laser condition (level 3) as PSTH plots, highlighting the estimated 50 % suppression of activity in the entire window condition consistent with previous reports (see Lee et al 2012), and transient suppression of activity in the early window condition.

Figure S10; related to Figure 5. Effects of PV cell activation on the activity of Pyr cells across the different time windows and task difficulty conditions. A) Histogram of stimulus selectivity (positive values: cells prefer rewarded go stimulus; negative values: cells prefer unrewarded no-go stimulus) for pre- and post-learning of the visual discrimination task. Pre: n = 1147, 10 mice, 0.05 ± 0.02; Post: n=1467, 9 mice, 0.14 ± 0.02; p < 0.002 (Wilcoxon rank-sum test). Median ± SEM. B) Average stimulus response activity at different times, ranging from 0 – 120 ms (laser duration), 120 – 240, 240 – 360, 360 – 480, and 480 – 600 ms, during the early window stimulation (0 – 120 ms) when stimulating PV cells with highest laser (control subtracted). Error bars, SEM. C) Average responses of two population of neurons (left panel, Pyr cells; right panel; PV cells) to optogenetic laser illumination at three different levels during the entire stimulus duration (0 – 2 s). The activity to the visual stimulus of Pyr cells (n = 1156) is suppressed, whereas PV cells (n = 60) is increased. Shading, SEM. D) Difference in mean visual stimulus-evoked response with PV cell activation (control subtracted), aligned to visual stimulus onset (continuous line). Cells were ordered by their averaged activity at 0 – 1 s. Colour bar range indicates activity, with positive values (blue) for cells increasing their activity and negative values (red) for cells decreasing their activity as a result of laser stimulation. E) is same as D) but for difficult task. F) Average activity response to visual stimulus in the absence (black) and presence (red) of laser stimulation, for the entire (left panel) and the early window condition (right panel). G) is same as F) but for difficult task. In plots D) to G): dashed line at 2 s for the entire window and at 0.12 s for the early window marks the laser stimulation offset; data shown for the highest laser power.

Why do so many more cells prefer the rewarded stimulus? Is this something intrinsic about the orientation selected, a consequence of training**,** or something more dynamic during the testing?

We thank the reviewer for the questions. We have now analysed and included new data, where we have quantified the median of the stimulus selectivity before and after learning (where positive indicates a higher preference for the go-stimulus and negative indicates a higher preference for the no-go stimulus). Thus, this effect is consistent with the bias being a consequence of training (see Poort et al 2015, Neuron; Khan et al 2018, Nat Neuro). We have now added this result to the manuscript in Supplementary Fig. S10A. We have updated the text in section ‘Changes in neural selectivity reflect behavioural performance in easy but not difficult discriminations’.

A) Histogram of stimulus selectivity (positive values: cells prefer rewarded go stimulus; negative values: cells prefer unrewarded no-go stimulus) for pre- and post-learning of the visual discrimination task. Pre: n = 1147, 10 mice, 0.05 ± 0.02; Post: n=1467, 9 mice, 0.14 ± 0.02; p < 0.002 (Wilcoxon rank-sum test). Median ± SEM.

* Likewise with Figure 6 and the model, which creates a few discrete populations of neurons preferring go or no-go stimuli and introduces competition via "strong" mutual inhibition. Activation of a second class of inhibitory "PV" cells which synapse only on excitatory cells and drive down their activity. The model, as constituted, does capture broadly some aspects of the neural data from figure 5. However, the model is not particularly well motivated or explained in the text of the manuscript making it difficult for me to accept that it is a reasonable model of what is going on in V1. Some specific questions I had that the manuscript doesn't answer: Are the Go/No-Go preferring cells in the same model are different? If different, why? Am I to interpret the Go-preferring input as reflecting orientation selectivity? Why is competition weaker between "other neurons" and No-Go preferring cells than G cells? Why do the PV cells only target Pry neurons and not other inhibitory
cells as in V1? How were any of the parameters chosen and is the model sensitive to the specific parameterization? This model is very simplified compared to the actual V1 circuits, I need more explanation of how/why the simplifying assumptions were made and how dependent the conclusions are on these decisions.

Q: Are the Go/No-Go preferring cells in the same model are different? If different, why?
Response: In our model, Go- and No-Go-preferring cells are identical in all intrinsic properties. The only difference lies in their connectivity. Specifically, Go-preferring cells are embedded in a circuit with stronger lateral competition compared to No-Go-preferring cells. This asymmetry was introduced to reproduce the experimentally observed differences in how these two populations respond to PV activation. To clarify this, we added to the methods section ‘Circuit model’ before introducing the values for the weights.

Q: Am I to interpret the Go-preferring input as reflecting orientation selectivity?
Response: In the model, we do not model orientation selectivity explicitly; however, Go-preferring cells receive higher input during the Go stimulus and lower input during the Nogo Stimulus and vice versa for the Nogo-preferring cells. This indicates a preference for the particular stimulus. Our model does not make any assumption about whether the Go or Nogo-preferring neurons were selective to the respective orientation of their preferred stimulus before training.

Q: Why is competition weaker between "other neurons" and No-Go preferring cells than G cells?
Response: Thank you for this question. The asymmetry in competitive connectivity was introduced to reflect the experimentally observed differences in how Go- and No-Go-preferring excitatory cells respond to optogenetic stimulation. In the model, this stronger competition modifies the stable activity states of the circuit, enabling more pronounced and long-lasting changes in Go-preferring cell responses following transient PV activation. This minimal asymmetry was sufficient to reproduce the differential effects observed in the data. We now clarify this rationale in the revised Results section ‘Circuit model captures changes in neural selectivity with PV cell activation’.

Q: Why do the PV cells only target Pry neurons and not other inhibitory cells as in V1?
Response: We appreciate the reviewer’s observation. In our model, we included only pyramidal and PV cells in order to keep the circuit minimal and analytically tractable, focusing on the essential mechanisms required to explain the observed effect. As such, there are no other inhibitory cell types in the model, and PV neurons target only pyramidal cells. This simplification was intentional: adding further interneuron subtypes and their interactions would introduce complexity that is not constrained by our data and, in our view, not necessary to account for the experimental findings. We now clarify this modeling choice in the Result section ‘Circuit model captures changes in neural selectivity with PV cell activation’ and Discussion sections.

Q: How were any of the parameters chosen and is the model sensitive to the specific parameterization?
Response: Thank you for this important question. The model was intentionally designed to be simple and interpretable, with only a small number of free parameters. In total, there are just six weight parameters, most of which were fixed at a value of 1. Only two of these, the inhibitory connections from PV to PYR, were adjusted to the values sufficient to reproduce the experimentally observed effects. The model is not highly sensitive to the precise values of these parameters. Rather, the key requirement is that certain qualitative connectivity constraints are satisfied (i.e. sufficiently strong PV-to-PYR inhibition). We now clarify this briefly in the Results (see the excerpt from the Results in the last comment).

We agree that the model is a highly simplified version of V1 circuitry, and this was a deliberate choice. Our goal was not to reproduce the full biological complexity of V1, but to isolate the minimal circuit components necessary to account for the observed experimental effect. By focusing on just pyramidal and PV cells, we aimed to clarify the computational mechanism without the confounds of additional, less-constrained parameters. The conclusions we draw from the model rely not on the exact structure of V1, but on a small set of core circuit motifs: specifically, competitive and PV-mediated inhibition. Our findings are robust as long as these qualitative connectivity features are present. We now more explicitly justify our modeling choices in the revised Results ‘Circuit model captures changes in neural selectivity with PV cell activation’ and Discussion sections.

* There should be some consideration/discussion of how plasticity, specifically SRP (e.g. Cooke and Bear J. Neuro 2010), might impact task performance. The training period is almost certainly driving SRP in your animals and changing the perception of familiar patterns. This plasticity is known to effect behavior in an orientation-dependent manner (see the "vidget") with a specific requirement for PV cells (Kaplan et al Elife 2016). Particularly relevant to your work, the consequences of plasticity on single cell firing are especially evident in the late sustained response period in SRP and also in a methodologically similar sequence learning task (Price et al Cereb Cortex 2024).

We thank the reviewer for the suggestions. We have now included this in the discussion.

Minor:

Fig S1A - the three vertically arranged rows seem to indicated changes with training but this isn't specified in the caption or labeled

We have now corrected this by adding labels to the plots to indicate the stage of training: early, mid or late.

A) Changes in licking and running speed profile over training (early, mid, and late) in an example mouse. Licks (black crosses) are aligned to stimulus onset in rewarded ‘go’ trials (blue shading, left panels) and unrewarded ‘no-go’ trials (red shading, middle panels). Red dots, reward delivery triggered by licks during the response window; yellow dots, reward delivery following auto-reward trigger. Average running speed (right panels) aligned to stimulus onset, for rewarded ‘go’ and unrewarded ‘no-go’ trials for the same example sessions. Shading, SEM.

Fig S1E - what is the running behavior here? Following my comments above about rebound activity, I'd like to see if the mice modulate their running after 2 seconds for the high laser power level.

Similarly to the licking plot, we have now added the running traces for this dataset to the supplementary figures (Fig. S4C). During the control condition, mice clearly detect the visual stimulus by rapidly reducing their running speed before licking to collect a reward. There is no such clear effect after the onset of PV activation (at 0s). There is a small dip in the running after the offset of PV activation (at 2s), which may be a response to the release of inhibition. However, this effect is small compared to the control. As discussed above in our response regarding Figure S1 E (now updated Fig. S4B), such rebound effects mainly occur at longer duration of stimulation and higher powers (see Li et al 2019). Importantly, we only analyse data from 0 - 2s (during stimulus presentation), where any possible rebound effects do not affect the outcome and interpretations. Furthermore, we show above that we do not see rebound effects for the early window, and most importantly, the behaviour improvement comes from a reduction in false alarms, not from an increase in licking. We have updated the text in Results section ‘Strong activation of PV cells in V1 impairs discrimination performance.

Running speed aligned to stimulus onset (black) or laser stimulation onset (colour). Based on their licking and running behaviour, mice cannot detect the laser stimulation alone during the 2 s period in the absence of visual stimulus. Vertical continuous line, stimulus or laser onset; vertical interrupted line, stimulus or laser offset.

# Reviewer #2:

The manuscript by Kukovska et al. addresses the question of how inhibition shapes the activity of V1 as a function of the timing and intensity of inhibition itself. The manuscript addresses an important question in systems neuroscience and is generally very carefully done. I only have a couple of comments that I think the authors need to address.

We thank the reviewer for their positive feedback and their constructive comments.

MAJOR

1) I have some trouble understanding the logic of the circuit model introduced in Fig. 6. The authors in fact do not justify how the model architecture was selected, or how it related to the actual cortical microcircuitry, in which multiple neuronal subtypes (and not only Pyr and PV+ neurons) form a complex network that actually implements the experimentally observed results. Given the apparently arbitrary nature of the model, I recommend that the authors either remove this part of the manuscript or replace it with a biologically plausible model incorporating different cell types, layers, etc. Given that a few of such models are available open source, this appears to be feasible, but I leave it up to the authors to decided whether to add a major modeling component (likely more suitable for a different manuscript) or instead expand the Discussion section with a section on the possible circuit-level mechanisms enabling the realization of the observed effects.

We thank the reviewer for this thoughtful and important comment. We fully agree that cortical circuits involve a diversity of inhibitory cell types and complex laminar organisation. However, our modeling goal was not to reproduce this full biological richness, but to construct a minimal circuit that captures the essential ingredients required to explain the experimental effects observed in our data. The model’s simplicity is intentional and serves a specific purpose: to isolate the key computational principles underlying the observed increase in selectivity following transient PV activation. Including additional cell types or laminar structure, in the absence of data from these populations in our current study, would make the model substantially more complex without being meaningfully constrained. Moreover, interlaminar and subtype-specific connectivity in mouse V1 is still incompletely understood, especially with respect to dynamic inhibitory control. Adding these components would risk introducing assumptions that are not justified by our data or other available data and could obscure the mechanisms we aim to highlight. In response to the reviewer’s feedback, however, we have introduced a more biologically grounded feature into the model: we now include explicit PV cell populations with their own activity dynamics, rather than modeling inhibition only through static recurrent inhibitory connections between pyramidal cells. This change improves the biological plausibility of the model while preserving its interpretability and computational clarity. We have clarified the rationale behind the model architecture in the Results section ‘Circuit model captures changes in neural selectivity with PV cell activation’, and we expanded the Discussion section.

Our focus here was to demonstrate that the experimentally observed effects can already be accounted for by a compact and analytically tractable circuit motif, which helps to distil the mechanisms at play. We hope this revised explanation and model update address the reviewer’s concerns and clarify the purpose and scope of the modeling work.

2) Related to the previous point, the Discussion section does not address the issue of how the observed experimental effects might be mediated by microcircuit-level mechanisms (e.g. other interneuronal subtypes, layers, etc.). This is an important aspect that should be addressed.

We have now added a paragraph to the discussion section motivating the model design. As mentioned above, given that interlaminar and subtype-specific connectivity in mouse V1 remains incompletely characterised, we deliberately decided to construct a minimal circuit that captures the essential ingredients to explain the observed experimental effects.

MINOR

1) The authors chose 120 and 180 ms post stimulus onset as time points for their optogenetic inactivation. While I think it's an elegant and parsimonious approach, the question remains about the precise time point at which the effect of inactivation will switch from an "early" to a "late" motif. However, I believe that the authors' definition on the early window might be too simplistic. In fact, several studies indicated how the requirement of V1 for visual processing varies as factors such as the complexity of visual stimuli, but also task complexity itself. The authors base their choice of window only on a couple of papers, but a discussion of whether the duration of the early window might be fixed or dependent of other factors (i.e. determined purely by fixed circuit-level architecture or instead dynamically adjusted by ongoing cortical processing) should be discussed, if only to point out the fact that the actual duration of the "early" window might co-vary with factors
such as task difficulty (something that was not explored in the present study).

We thank the reviewer for the comment. We have now added a section in the discussion to address these points. We acknowledge that the precise timing of the early and late phases is likely dependent on various factors such as stimulus and task complexity. We had to choose a limited set of time windows, since we would otherwise have too many experimental conditions (with multiple power levels, time windows and difficulty levels) resulting in too long experimental durations for the animals. We based our chosen timings and boundary between the early and late phases on previous literature in the mouse (Resulaj et al 2018, eLife; Javadzadeh and Hofer 2022, Neuron).

Additionally, we would like to highlight our results in Supplementary Fig. S8 (previously Supplementary Fig. S3D-F). We investigated the effect of changing the duration of the early window during difficult discriminations, and extended it to 180 ms or 340 ms. However, we found that the changes in d’, hit and FA rates were comparable to the standard early window ( 0-120 ms). Thus, we did not identify manipulations in the strength or timing of PV cell activation that improved performance in difficult discriminations even for extended time windows. However, we agree with the reviewer that the effect of variation of timing of stimulation will be interesting to further investigate in future studies.

Figure S8; related to Figure 3. No behavioural improvements in difficult discriminations by extending the early window beyond 120 ms. A) Normalized average discrimination performance, d’, B) hit rate, and C) false alarm rate as a function of laser power for the original (0 – 120 ms) and two new (0 – 180 ms and 0 – 340 ms) ‘early’ windows (N = 4 sessions). Data is normalized by subtracting the value of the 0 power level (see Methods). Shading, 95% CI of bootstrap distribution.

2) The plots presented in Fig. 5F-G are unclear. While a shift in preference of neurons can be seen, the chosen display makes it difficult to appreciate the magnitude and significant of this shift. A method enabling a quantification of this shift would be preferable.

We thank the reviewer for this comment. In the previous version of the manuscript, we quantified the shift towards more go-preference by comparing the median selectivity (positive indicating go-stimulus preference, negative no-go preference) in the control and with laser stimulation for the easy condition. We have now added the quantification for all other conditions to Figure 5 legends and further clarified the description in the text.

Easy task, Entire: median ± SEM, Control = 0.16 ± 0.02, Laser = 0.18 ± 0.02, p = 0.000, N = 1156

Easy task, Early: median ± SEM, Control = 0.18 ± 0.02, Laser = 0.31 ± 0.02, p = 0.000, N = 754

Difficult task, Entire: median ± SEM, Control = 0.15 ± 0.02, Laser = 0.12 ± 0.01, p = 0.931, N = 542

Difficult task, Early: median ± SEM, Control = 0.15 ± 0.01, Laser = 0.10 ± 0.01, p = 0.000, N = 561

# Reviewer #3:

Kukovska et al., investigate how parvalbumin-expressing inhibitory neurons (PV) influence visual discrimination in mice. The research primarily examines how varying both the activation strength and timing of PV neurons affects visual orientation discrimination across two difficulty levels.
The manuscript is clearly written and the conclusions will be of wide interest to the community. Overall, I believe that most experiments were well-conceived. However, several areas would benefit from additional controls and/or expanded discussion to strengthen the interpretation of results:

We thank the reviewer for these supportive statements and their helpful comments.

Major concerns:

1) The extent of optogenetic driving of PV cells under different laser powers is not established or self-evident from the data presen The change in PV cell activity and the corresponding change in  Pyr cell activity across laser intensities should be quantified, and ideally verified with electrophysiology to have a meaningful metric of spike output from PV cells under optogenetic drive. Additionally, since PV cells are distributed throughout all layers, varying the laser power can selectively activate PV cells at different depths. These aspects need to be tested and clarified to have solid conclusions about the role of PV cell on visual discrimination.

Similar to previous studies, we have aimed to suppress the population of Pyr cells by around 50 % (see supplementary of Lee et al 2012, Nature). We have now plotted the z-scored dF/F averaged across all cells in the dataset (Pyr = 1156; PV = 39) in Supplementary Figure S10C, and show that the example cells in Fig 5C are representative of the population.

A recent study quantified the laminar profile of PV activation using optogenetic illumination of the cortical surface as was used in our study (Li et al., 2019, Elife). They found that inhibition was relatively homogenous and extended across cortical layers, ranging from L1-L5/L6 (their Figure 4A and B). Furthermore, they show for the laser level that is closest to the range that we use, a relatively homogeneous activation strength of fast spiking interneurons (their Figure 4C). Importantly, in our study we established that the behaviour effects were consistent across a range of laser powers. We have added these considerations to our discussion.

C) Average responses of two population of neurons (left panel, Pyr cells; right panel; PV cells) to optogenetic laser illumination at three different levels during the entire stimulus duration (0 – 2 s). The activity to the visual stimulus of Pyr cells (n = 1156) is suppressed, whereas PV cells (n = 39) is increased. Shading, SEM.

2) The conditions of the experiments make extrapolating to the "normal" function of the cortex, i.e. visual circuits under more naturalistic sensory inputs. While this is hardly a novel criticism and a fault common to all our experiments, it should be addressed in the discussion. For example the experiments use full field visual stimulus which reduces drive on both Pyr cells and PV cells from SST neurons, a significant confounder in estimating the output of PV cells in these experiments (and also why concern 1 is so critical). Additionally optogenetic driving of PV cells will have distinct population effects to how PV cells operate in the visual cortex, with almost all PV cells being equally driven at nearly simultaneously. This may very well negate established cortical circuits for sharpening feature selectivity such as lateral inhibition in a winner-takes-all circuit. Along a similar vein, the choice to explore difficulty along the dimension of angle discrimination rather than say contrast also affects to which visual tasks the result are applicable to. These issues should be acknowledged in the discussion.

We thank the reviewer for this suggestion. We have now added sections in our discussion about these points.

In this study, we used full-screen visual stimuli to enable homogenous visual input to drive visual cortical neurons in our imaging field and facilitate comparison with previous studies using similar stimuli (e.g. Lee et al 2012, Nature; Poort et al 2015, Neuron). We have not compared the effect of using smaller stimuli. It is indeed possible that the effects of PV activation are modulated by stimulus size since it may influence the amount of surround suppression and relative recruitment of specific interneurons (Adesnik et al 2012, Nature) and this will be an interesting direction for future studies.

Second, in this study we use optogenetic activation of all PV interneurons in the field of stimulation. One of the reasons that we think are results are interesting is that it is not necessarily predictable that this can improve behaviour or enhance selectivity. Therefore, the fact that this does indeed improve behaviour and selectivity is important to report, also because it would facilitate future applications to boost behaviour and neuronal selectivity without requiring detailed knowledge of feature preferences of individual cells. Our results delineate the conditions under which PV activation can benefit performance dependent on laser level, timing and task difficulty. Another interesting direction for future research would be to determine to what extent the behaviour and neural effects rely on specific cells within the imaging field, for example by using spatial light modulators for two-photon photostimulation to activate subpopulations of cells (see for example Russell et al 2024; Nature Communications).

Third, we have added motivation why we use experimental manipulation of orientation difference to change task difficulty. Cells in the primary visual cortex are known to exhibit strong orientation selectivity (Niell et al., 2008). Primary visual cortex is known to be necessary for orientation discrimination (e.g. Glickfeld, 2013, Poort et al. 2015). Manipulating the orientation difference is a widely used approach to manipulate task difficulty (e.g. Lee et al., 2012; Resulaj et al., 2018; Lyamzin et al. 2021) facilitating comparison with previous work. We agree that other kinds of manipulation (e.g. contrast, spatial frequency) could differentially affect neural activity and therefore the effect of PV activation; it would be interesting for future studies to compare the effects of other types of manipulations.

3) It is difficult to compare the effects across different cohorts of mice that use different genetic strategies for optogentic expression. For the 2P experiments to be relevant to previous PV-Cre ChR2 experiments the author must establish the spread and penetration of their viral strategy. The examples shown in Fig 5B clearly show ChrimsonR labelled PV without any detectable GCaMP expression. How many of all the PV cells expressed ChrimsonR and GCaMP? How far was the viral spread in V1?

In response to the reviewer question, we have now added estimates of the spread of GCaMP and ChrimsonR. As described in the methods, viral injections were made at 2 injection sites (coordinates ML2.5 and ML2.3) at depths of 200 μm and 400 μm from the brain surface and labelled cells across all 6 V1 layers (Fig. S2E). Our in-vivo imaging data and histology indicates that the spread of GCaMP and Chrimson fluorescence is approximately 800 microns and across V1 layers 1-6 (Fig. S2E-G).

We also clarified that the example shown in Fig 5B shows an average of 150 frames in the middle of the recording (corresponding to 5 seconds), therefore not all cells in the field of view will be active simultaneously. We also added new immunostaining data to quantify the percentage of PV cells expressing ChrimsonR-tdTomato and ChR2-EYFP (see Fig. S2A-D).

Minor concerns

a) In Figure 5 simple deltaF traces of individual responses which include the end of the visual stimulus would improve the figure and help reassure the reader.

We have now updated the plots.

b) In the introduction the authors write " By innervating Pyr cells at the soma and axon initial segment, PV cells are strategically positioned to facilitate neuronal processing ", this is nonsense as all inhibitory synapse (and really all synapses) are in a position to facilitate neuronal processing.

We have now rephrased this sentence: “PV cells innervate Pyr cells at the soma and axon initial segment [15,16], and are on average the strongest contributor to inhibition onto Pyr cells (Pfeffer et al. 2013)"

c) Figure 5F was difficult to read, the color bar scale needs to be much larger and easier to read

We have made the scale bar larger and increased the font size.

d) Examples of the V1 / HVA maps from the Intrinsic imaging should be included in the supplemental figures.

We have now included an example intrinsic imaging map in relation to the mask used for the primary visual cortex (Supplementary Fig S3).

e)The text in most figures is extremely small and difficult to read. Font size should be increased and standardized throughout the manuscript.

We have corrected the font size in all figures throughout the manuscript.
