# Supplementary material for: Context-dependent activation of V1 parvalbumin interneurons enhances visual discrimination
Source: PLoS Biol. 2025 Nov 25;23(11):e3003518. doi: 10.1371/journal.pbio.3003518 (PMC12677792; doi:10.1371/journal.pbio.3003518)
Supplement: Attachment — Submitted filename: ResponseToReviewers_Revision2.docx [file pbio.3003518.s015.docx]

# Response to reviewers:

# Reviewer #1:

Reviewer #1: Overall, the authors have been diligent in addressing and responding to my questions and critiques. I particularly appreciate the inclusion of new supplemental figures, particularly S10, which provide significantly more insight into the methodology and underlying neural activity. Likewise, the extended discussion better situates the work in the broad context of other studies and I have no major concerns with the data as presented.

We thank the reviewer for their supportive and helpful comments and suggestions.

Minor issues:

The highly simplified model of cortical circuits now explicitly represents a deliberate choice to demonstrate that effects similar to those seen in the data can be achieved by a particular circuit motif which is relatively insensitive to parameterization (fig S11). While the motivations for the model are now more obvious, and the simplifying decisions fit generally within computational norms, I remain skeptical that a model that is so reduced relative to the biology tells us much about underlying mechanisms in this case, particularly given it's reliance on a bespoke motif that is not well motivated by biological observations and completely ignores how other GABAergic populations could effect the dynamics of PV and PC firing. If the overall impact of the paper hinged on this I would not recommend publication without significantly more work extending the model or validating it in vivo. As it is, the experimental data speaks for itself and the model feels like a relatively inconsequential add-on suggesting one possible mechanism without convincingly demonstrating it. I don't want this to sound overly harsh, it is a a clever idea and I would be interested to read a computational work by the same group investigating how this motif functions within a more realistic dynamic environment, or that explains how the required asymmetric strong/weak competition circuits form in the first place, or makes testable predictions subsequently validated experimentally. In its current form, however, the model does little except say, essentially, "if PC cell firing is dominated by a small subset of GABAergic cells that are wired together in a very particular way, that could explain one aspect of our experimental observations".

We appreciate the reviewer’s thoughtful comments on the scope of our model. We fully agree that a more mechanistically detailed model (including the diversity of interneurons, circuit formation mechanisms, richer network dynamics) would be an important next step toward a more mechanistic understanding. However, developing and validating such a model would constitute a substantial modelling study in itself and would not be sufficiently constrained by the currently available data.

Our recordings include only excitatory and PV cells, and comparable data for other inhibitory subtypes are not yet available. Moreover, quantitative information on the connectivity between specific subpopulations in mouse V1 remains incomplete: the literature provides only population-level statistics rather than the detailed connectivity. Under these conditions, a more complex model would need to make arbitrary assumptions and potentially complicate the identification of the circuit mechanisms our current data can directly constrain.

Our goal here was, therefore, to use a deliberately simplified model to demonstrate that a specific type of circuit motif is sufficient to reproduce the qualitative effects observed experimentally. We see this as a conceptual bridge between the experimental data and more detailed future modelling studies, rather than as a definitive mechanistic account of the underlying circuitry.

We revised the manuscript to clarify this intent and included more explicitly the limitations, and potential extensions for future modelling studies, and what experimental data would be required (see Discussion).

# Reviewer #2:

We thank the reviewer for their positive feedback and their constructive comments.

1) In their rebuttal the authors provide a supplementary figure to clarify what they displayed in Fig. 5F-G. I think that this figure more transparent and clear in presenting the data and the small (yet significant) size of the effect the authors report. I strongly recommend to replace Fig. 5F-G with the panels that the authors included in their rebuttal. The statistics (values and types) should be included in the legend of the updated Fig. 5F-G.

Thank you for the suggestion: we have now added the panels to the figure.

2) The updated computational model is a significant upgrade. As a minor point, I suggest that the authors harmonize the y-axes in Fig. 6B-F, so that it is clearly visible what the different between the various conditions are. I was also rather surprised by the fact that the updated model yielded results that were basically undistinguishable from the earlier model (error bars may be slightly different, while the average values appear unchanged). I wonder if the authors have an explanation for this, or if it is just an aesthetic effect.

We thank the reviewer for these two valuable comments. First, we harmonised the y-axes as suggested. Second, the similarity between the updated and earlier model results arises because, despite the explicit inclusion of a rate-based inhibitory population, the effective circuit dynamics remain nearly equivalent to the previous implementation. In the earlier version, inhibition was modelled as a direct negative coupling term between excitatory units. In the revised model, inhibition is mediated by an explicit interneuron with simple linear dynamics.

$$\tau^{I}\frac{dr_{i}^{I}}{dt}=-r_{i}^{I}+W^{I,E}r_{i}^{E}$$

This formulation introduces a small temporal lag in inhibitory feedback but does not qualitatively alter the steady-state relationships between excitation and inhibition. After extending the simulation time to allow the system to reach equilibrium, the network converges to similar fixed points as the original, more abstract formulation. This explains why the results of the new version look similar.

# Reviewer #3:

Based on the authors' responses to my questions as well as those posed by other reviewers, all of my concerns have been adequately addressed. Thank you.

We thank the reviewer for their helpful comments for our revision.
